# Why we need a process on breaking news of Juvenile Idiopathic Arthritis: a mixed methods study

**DOI:** 10.1186/s12969-016-0092-6

**Published:** 2016-05-21

**Authors:** Aurélie Chausset, Anne-Laure Gominon, Nathalie Montmaneix, Stéphane Echaubard, Séverine Guillaume-Czitrom, Benoit Cambon, Cécile Miele, Emmanuelle Rochette, Etienne Merlin

**Affiliations:** Pédiatrie, CHU Estaing, Clermont-Ferrand, France; Pédiatrie, INSERM-CIC1405, CHU Estaing, 1, place Lucie & Raymond Aubrac, 63003 Clermont-Ferrand, cedex 1 France; Service de Médecine des Adolescents, CHU Bicêtre, Le Kremlin-Bicêtre, France; Département de médecine générale, Faculté de Médecine, Clermont-Ferrand, France; CRIAVS, Pôle Santé Publique, CHU Gabriel Montpied, Clermont-Ferrand, France

**Keywords:** Juvenile idiopathic arthritis, Parent’s experience, Diagnostic counseling, Doubt, Qualitative research

## Abstract

**Background:**

Juvenile Idiopathic Arthritis is the most common chronic pediatric rheumatic disease. The announcement of Juvenile Idiopathic Arthritis poses for parents a number of challenges that make it hard to accept a diagnosis of the disease for their child; yet to our knowledge, no study to date has focused on the time period immediately surrounding the diagnosis. This study sets out to describe parents’ experiences in engaging with their child’s diagnosis of Juvenile Idiopathic Arthritis.

**Methods:**

This is a mixed methods study. Semi-structured interviews of families with a Juvenile Idiopathic Arthritis child were conducted. A grounded-theory thematic analysis was performed. Items that emerged in the interviews were compiled into a self-administered questionnaire.

**Results:**

Eleven families participated in the qualitative study. Sixty families responded to the questionnaire. The path of parents was characterized by doubt (before, during and after diagnosis) while the disease tended to take center stage. Doubt was generated through mismatches in perspectives between the parents’ circle of acquaintances, physicians, and the parents’ own subjective experiences of symptoms. This study also found that social support and parent associations occupied an ambiguous position between help and stigmatization.

**Conclusions:**

Doubt fuels self-energizing spirals that take root as parents learn the news that their child has Juvenile Idiopathic Arthritis. These spirals of doubt may influence parents’ experiences at every stage throughout the course of disease. Our data support the implementation of a specific process dedicated to breaking the news of Juvenile Idiopathic Arthritis to parents.

## Background

Juvenile Idiopathic Arthritis (JIA) is the most common chronic pediatric rheumatic disease [1]. It is defined by the onset, before the age of 16 years, of inflammatory joint symptoms persistent for at least 6 weeks and of unknown cause [2]. The term JIA actually encompasses a heterogeneous group of different diseases classed into 7 categories of variable severity and long-term consequences depending on symptom manifestations and response to treatment. JIA qualifies as a rare disease as it affects somewhere between 16 and 150 of every 100,000 children [3], which is part of the reason General Practitioners (GPs) and the general public fail to understand that rheumatism can affect children too.

A diagnosis of JIA also poses a number of challenges for parents that make it hard to accept a diagnosis of JIA for their child. First, the term “Juvenile Idiopathic Arthritis” does not refer to a single disease, but rather a cluster of diseases [4] that all follow different flare-and-remission patterns in patients. Objective symptoms are often invisible or seem much less severe than they really are, and the long-term consequences are hard to anticipate. Second, follow-up is essentially through out-patient consultations with a few short hospitalizations. Home-based care is generally provided by relatives. JIA is clinically managed by a multidisciplinary team. As patients are not frequently admitted to the hospital, follow-up takes place in a setting limited in both time and space, leaving little room for practitioners to perceive or understand the perspectives and experiences of the parents. JIA is also characterized by a long delay between the onset of symptoms and eventual diagnosis. This lag is highly variable and is sometimes made even longer when multiple medical intermediaries are involved. The net result is that the first actual disclosure of diagnosis may only come many months after the symptoms first emerge.

In many severe chronic diseases and disabilities, learning the diagnosis is described as a brutal event in which parental benchmarks are disrupted [5]. There are many studies addressing parents’ feelings (shock, stress, isolation, fear of the future) and coping strategies [6–9], but few studies focus on the impacts of experiences during the period of JIA onset and ever fewer on the period immediately surrounding the diagnosis of JIA. However, the announcement of a chronic rheumatological disease can trigger a lot of concern. In spite of this, to our knowledge, no study to date has focused on the specific moment when the diagnosis of JIA is announced to parents. To address this gap, we led this study to describe parents’ experiences upon learning that their child has JIA, in order to define cornerstones to help physicians implement a JIA-specific set of diagnostic disclosure measures.

## Methods

The project was approved by the interregional ethics committee (CECIC Rhône-Alpes-Auvergne, Grenoble, IRB 5921).

In this study, we used a multi-method approach. We first led a qualitative study to gain in-depth insight into parents’ experiences upon learning their child has JIA. We collected a wide field of items that were then compiled into a self-administered questionnaire on the diagnosis delivery interview.

A. Qualitative study
  - Patient selection
    Parents were eligible for inclusion if their child was:
    diagnosed as having JIA between 4 and 18 months before the start of the study.
    treated and followed at the pediatric rheumatology unit of Clermont-Ferrand university hospital or Bicêtre hospital.
    Participation in the study was not proposed to families when at least one of the parents presented health conditions incompatible with an individual interview. Families were selected to obtain a heterogeneous sample. Data were collected until data saturation (i.e. ‘theoretical saturation’, where the interviews no longer emerge any new or relevant data).
  - Methods

### Recruitment and data collection

Semi-structured in-depth interviews of parents were conducted between May 2013 and December 2014. Informed consent was obtained before each interview. All interviews were conducted by an investigator not involved in the patient’s care path. Interviews took place in a quiet room in the hospital. Using a predefined guide, the interviewer asked questions designed to probe the issues and clarify the statements.

In addition, data collection included gender, age, diagnosis (according to the International League of Associations for Rheumatology classification) and disease activity. Disease activity was defined as inactive when a patient met the following criteria: a physical global assessment reported as 0 by the pediatric rheumatologist; no joints with active arthritis; no active uveitis; no fever, rash, serositis, splenomegaly or generalized lymphadenopathy due to JIA; normal erythrocyte sedimentation rate; morning stiffness ≤ 15 min [10].

### Data analysis

All interviews were audiotaped and transcribed verbatim. Field notes were made during sessions. Grounded-theory thematic analysis (a methodological approach for developing theory that is “grounded in data systematically gathered and analyzed”) was performed [11].

A coding frame was constructed and refined during the progression of analysis. Two members of the research team coded the data. AC and ALG performed the first analysis, after which AC, ALG and EM triangulated the interpretations. Themes were organized into categories then concepts.

B. Questionnaire

In a second step, we constructed a study instrument building on themes that emerged from the interviews. The questionnaire was built by the pediatric clinical research team and tested with caregivers, psychologists and parents. It was divided into 4 parts:

- Diagnosis and initial follow-up
- Physician’s attitude towards the delivery of the diagnosis
- Following the announcement (other sources of information, explanations needed, appreciation of disease severity, feelings experienced)
- What is the parents’ idea of the “ideal delivery of diagnosis”?

The questionnaire includes an assessment scale with 4 levels (strongly disagree/disagree somewhat/agree somewhat/strongly agree), multiple choice questions and grading scale.

All children diagnosed with JIA and living in Auvergne were considered for potential enrollment in the Auvergne Loire observatory on JIA (APICALE observatory) wich has been in operation since 2011. All parents of children with JIA who were a part of this observatory and followed at the Clermont-Ferrand university hospital were invited to answer. The questionnaire was proposed separately to each JIA child’s mother and father. They also had the opportunity to complete the questionnaire together.

Study data were collected and managed using REDCap (Research Electronic Data Capture software build 5.7.4 ©, Vanderbilt University, 2014), a secure web-based electronic data capture application hosted at CHU Clermont-Ferrand [12]. Descriptive statistics were used to describe the population and the frequencies (median, mean, range).

## Results

A total of 11 families were ultimately included in the qualitative study, counting 11 mothers and 8 fathers (i.e. 8 couples and 3 single mothers). Children were 3 to 15 years of age and presented a variety of JIA subtypes and disease severities. Of the 80 families invited to complete the questionnaire, at least one completed questionnaire was obtained from 60 out of 80 families invited to participate (Table 1).

**Table 1:** Demographics and clinical characteristics of the sample

a) Qualitative study (n=11 patients)	
Diagnosis	Disease activity
Oligoarthritis	Inactive
Oligoarthritis	Inactive
Oligoarthritis	Inactive
Oligoarthritis	Active
Psoriatic arthritis	Active
Enthesitis-related arthritis	Active
Systemic arthritis	Inactive
Psoriatic arthritis	Active
Polyarthritis	Active
Unclassified arthritis	Active
Polyarthritis	Active
b) Self-administered questionnaire - Characteristics of the families as reported in the questionnaire	
Parents (Sample n=64)	n (%)
Mother	42 (66)
Father	22 (34)
Children (Sample n=60)	Range (median)
Current age (yr)	2-21 (13)
Age at the diagnosis of JIA (yr)	1-16 (8.5)

Three main themes emerged from analysis of the interviews: doubt, the prominent place of the disease in parents’ lives, and the need for medical counseling.

A. Doubt
  - Confusing factors before diagnosis
    Once symptoms appeared, the parents swung between trivialization and over-dramatization of symptoms. This uncertainty was enforced by mismatches between the “alarmist” circle of acquaintances (e.g., daycare staff, grandparents) and the “reassuring” medical profession. Indeed, most parents reported that the first doctors consulted initially provided explanations without giving a specific diagnosis. Their discourse was reassuring to parents as it associated the symptoms (pains, limp, tiredness) with the most common health problems in children (growth, maladjusted first shoes, sports injuries, and tendinitis) (Table 2 quote 1). The lack of satisfying explanations on the persistence of symptoms could lead parents to question the origin or real grounds of the pain (psychosomatic, pain as an excuse). This path could ultimately lead to “fantasy”, where parents tended to imagine the worst and focused on what scared them most, like cancer.

**Table 2:** Doubt: Quotes from parents

Quote 1: “We’d taken him to see the doctor, where they told us she maybe had pain in her toe, that it was maybe caused by her shoes, a whole bunch of things… then as the days went by and she still wouldn’t walk, so we took her to the emergency ward, because the daycare centre people, they were worried too, and then even at the emergency ward it takes ages—ages and ages—we spent whole days there, all without result because at the end they said it was maybe irritable knee, or irritable hip, so we left it there, we thought that was it, and we went home thinking that was it and it should be over in the next six weeks.”
Quote 2: “We had already been down every road we could. In fact, every day I was back home for early afternoon, so I’d spend my afternoons scouring the internet, running searches on her symptoms and trying to find out what she might have. On top of that, I’ve got a friend who’s a test-lab technician, and another friend who’s a nurse.”
Quote 3: “Well, materializing the problem, seeing what it is, knowing how to manage it, learning how to handle it, it’s like… like it cancels out some of the fear, some of the uncertainty.”
Quote 4: “You have to understand the name they give it—the name is pretty obscure, there’s always going to be a scientific term for it, but what people need is a simple name that’s easy to understand, or at least explanations straight away for each of the terms, so, yeah, the French lesson that goes with it.”
    In some cases, initial misdiagnosis could prompt moves on the part of parents to engage in their own active medical diagnostic approaches (with friends, the internet, making hypotheses and deductions), especially if physicians failed to bring satisfactory answers. This trend to substitute for the doctor implied an appropriation by the parents of the medical terms and concepts and also the treatments involved (Table 2 quote 2).
    Overall, in the time from onset to diagnosis, parents were going to experience doubt about themselves, the doctors, and their child, while sometimes adopting an overlapping role between parent and diagnosis-maker.
  - Delivery of the diagnosis does not dispel the doubt

After the phase where parents were left floating without a specific diagnosis, the moment of the diagnosis was both expected and feared. Diagnosis brought some structure but did not dispel all doubts and also brought new uncertainties.

### Shock and relief

The diagnosis put an end to this period of doubt, finally giving an explanation to the symptoms. At the same time, the parents discovered how ill their child was and how real the symptoms were.

In the self-administered questionnaire more than half of the parents (37 out of 64) reported that the announcement of JIA was a shock and most of them reported that they were concerned (51 out of 64). More than a third (23 out of 64) of parents reported that the announcement of the diagnosis was a relief (Table 3).

**Table 3:** Parent’s perception of the announcement of the diagnosis. Responses to the assessment scale questions

Sample *n*=64	*n* (%)
“What was the emotional impact of the announcement of the diagnosis on you?”	
It was a shock	
Agree strongly/Agree somewhat	37 (58)
Disagree strongly/Disagree somewhat	13 (20)
No answer	14 (22)
I felt guilty	
Agree strongly/Agree somewhat	27 (42)
Disagree strongly/Disagree somewhat	23 (36)
No answer	14 (22)
I was angry	
Agree/Agree somewhat	15 (23)
Disagree/Disagree somewhat	31 (49)
No answer	18 (28)
I was sad	
Agree strongly/Agree somewhat	30 (46)
Disagree strongly/Disagree somewhat	17 (27)
No answer	17 (27)
I was concerned	
Agree strongly/Agree somewhat	51 (80)
Disagree strongly/Disagree somewhat	4 (6)
No answer	9 (14)
I was relieved	
Agree strongly/Agree somewhat	23 (36)
Disagree strongly/Disagree somewhat	23 (36)
No answer	18 (28)
“Did you think that your appreciation of the severity of the disease was different from the physician’s?”	
Agree strongly/Agree somewhat	34 (53)
Disagree strongly/Disagree somewhat	23 (36)
No answer	7 (11)

The announcement was already difficult for the parents, but it became even more difficult to process when the parents felt out of sync with the doctors’ assessment of the situation at the time of diagnosis (for example: “indolent oligoarticular JIA” for the doctor could be understood simply as a “terrible chronic disease” for the parents). In the self-administered questionnaire, when asked “Did you think that your appreciation of the severity of the disease was different from the physician’s?” more than half of the parents (34 out of 64) responded that their perception was different (Table 3).

### Structure and confusion

The delivery of a diagnosis brought structure by: providing a clear path of care, referring to a specialist doctor, erasing any doubts as to the reality of pain, and discharging the parents from the medical process they had engaged in (Table 2 quote 3).

Apart from clearing the confusion, the delivery of the diagnosis carried new uncertainties.

First, the name of the disease could be disturbing (Table 2 quote 4). Parents found it was hard to understand and accept the term “idiopathic”. The lack of knowledge on the causes and mechanisms of disease induced guilt over what the parents should or should not have done. Some parents reported that they would have preferred that the doctor told them it was their fault, despite the guilt, rather than that the disease was due to some unknown cause.

Second, mid- to long-term evolution varies greatly between and within the different categories of the disease. Moreover, treatment options raised a lot of hope, but the price to pay was that treatments were often given reluctantly (because of fear of side effects combined with the age of the patients and the chronic nature of the disease). Indeed, although the diagnosis of a patent disease was established, parents (especially parents of young children) were asked to deal with the recurrent ambivalence between the strict “sick/not sick” dichotomy and the variable patterns in which the disease manifests (“sometimes sick, sometimes not sick”).

B. Parents as frontline caregivers
  - Struggle for normalcy
    The disease appeared like a “roller-coaster” that parents would attempt to smooth over. The “struggle to bring normalcy to their child’s life” took parents out and away from the “normal life as parents”. Parents of “new-onset chronically-ill children” hovered between the world of the disease and the normal world (Table 4 quote 1). To deal with the suffering and solitude generated by the disease, parents looked for support, first and foremost from friends and family. However, social support brought more blurred lines: it was made necessary by living with the disease, but would take parents still further away from “normal life” and towards a “disease-driven life”. This could explain the reluctance of parents to join disease associations or seek counseling, as it could confine them into the “disease-driven life” (Table 4 quote 2).

**Table 4:** Parents as frontline caregivers: Quotes from parents

Quote 1: “So curiosity got the better of us and we had a look at the website, to see if they gave a bit more info, but me, all I really saw was getting definitively sucked in, wrapped up in the world of the disease.”
Quote 2: “What actually scared me the most was that there was this association, an association of parents to deal with the disease, and I told myself no way am I going down that road, because for the minute she’s looking OK so I need to stop myself seeing the disease.”
Quote 3: “Basically, from then on, any move she makes, you're on the alert, because you're looking out for whether she is responding to the drugs, because she’s still too small to be able to tell you whether or not the treatment is effective. So, you know, with that, at the slightest little thing…”
Quote 4: (on psychological counselling) “More for his little brother, I would think— because we've no idea how all that might have affected him.”
  - Parents as caregivers
    As soon as a diagnosis was made, the parents found themselves central to disease management (treatment administration, monitoring side-effects, detecting new symptoms, signs of flare, transmitting medical information to their medical team, and liaising with the referring physician) (Table 4 quote 3). The parents were often reluctant to deliver some of the drugs (seen as ineffective or in some cases likened to poison) to their child. Aside from this permanent adjustment to the disease’s fluctuations, some parents attempted to prevent the possible side-effects of the treatments by adopting a safer lifestyle, often by modifying their food habits or sometimes by using complementary medicines (homoeopathy, herbal medicine).
  - Everything organizes around the disease
    The parents were necessarily active in the care path, and this activity drove them to devote a great deal of time to the disease and to acquire new competencies. As a consequence, the disease took a central place in their social links, and their environment was perceived through this new activity that now dominates their lives: friends interested in the disease shared the main field of interest of parents. Many of the parents’ social relationships were therefore organized around the disease.Since the family’s whole life was connected to the disease, a distinction appeared between the family’s children: the healthy sibling’s life was not neglected but tended to be organized around constraints tied to monitoring the ill child. The parents recognized the need for explanation and consideration of the sibling, but without giving priority to psychological support (Table 4 quote 4).

C. Medical support: needed but lost
  When it came to managing the disease, parents were left to fend for themselves. Parents were vulnerable on two fronts as they were forced to contend with two new roles: parents of a newly-ill child, and inexperienced caregivers. In this period of uncertainty, the parents often felt frustration and distrust towards health professionals. After the delay in diagnosis and loss of confidence in GPs, the pediatric rheumatologist tended to become the main referent doctor. However, parents also felt that their voice is not heard and their difficulties and concerns are not understood during the diagnostic progress (by GPs, pediatricians, and paramedics) (Table 5 quote 1).

**Table 5:** Medical support, needed but lost: Quotes from parents

Quote 1: “I do think, though, that a diagnosis, to get it right, you have to ask the right questions, and you have to know how to really listen to you—and a doctor that doesn’t know how to do that, to really listen, shouldn’t be a doctor, he should do something else, anything else, just not doctor—I think that at the end of the day, the diagnosis hinges on you as the patient, you know your body, if A says he’s in pain now, then they should’ve listened to us.”
Quote 2: “Online you’ve got the whole gamut, every extreme—you find people who tell you yes, you can beat it, that it’s over soon enough, or rather not soon enough, that you can battle through it, and then you find people who’ve been through nothing but heartbreak and anguish because of the disease, heartbroken for their child, and… you wonder how you're going to be on that scale.”
  Since the announcement capped a tortuous path with a sharp and brutal event, parents often left the first consultation staggered and shocked, which means much of the information delivered at that point did not get fully assimilated. Moreover, many questions often emerged after the end of a consultation, prompting many parents to turn to the Internet to look for information during two key periods:
  The period before diagnosis (to find explanations of symptoms, looking for a diagnosis)
  In the wake of the diagnosis announcement (to try to understand the disease and the medical terms used).

**Fig. 1:**
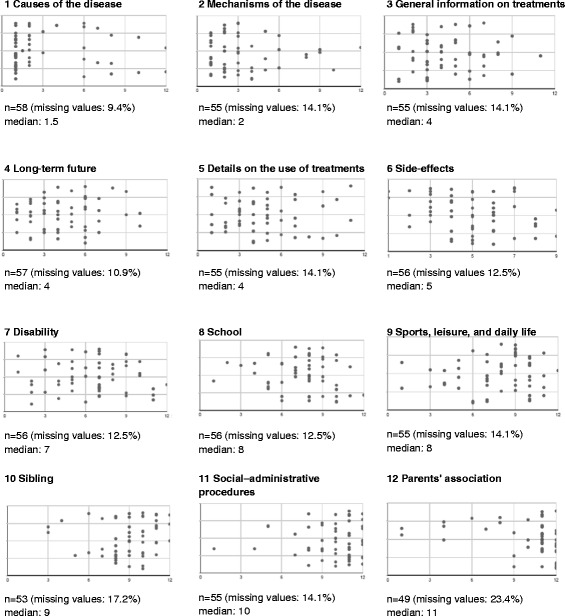
Ranking of items to be approached during the process of announcing JIA, in descending order. Distribution of parent’s responses in answer to the question: “In your opinion, which themes must be approached first and foremost during the initial diagnosis communciation consultation? Rank them from 1 to 12”

The need for a better understanding of the disease was a key point for the parents. In the self-administered questionnaire, when asked “In your opinion, which themes must be approached first and foremost during the initial diagnosis communication consultation?” parents responded that causes and mechanisms of JIA were important topics to be addressed, while items concerning everyday life or social and administrative support were given the lowest priority (Fig. 1). However, parents needed to get information about resources and support. This might be good evidence of the need for a two part announcement process, first addressing causes and symptoms, then later addressing resources and support.

Parents were critical about the Internet, yet most parents had gone online to find information. Official sources of information were privileged over testimony-type websites and forums that were deemed disruptive and frightening (Table 5 quote 2).

## Discussion

This study set out to describe parents’ experiences upon learning the news that their child has JIA. Our study finds that the path of parents of a child who gets diagnosed with JIA was characterized by an absence of benchmarks. In many circumstances, the parents were exposed to ambiguities or ambivalences. Doubt was a key feature of engaging with JIA.

**Fig. 2:**
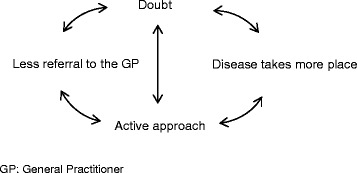
Self-energizing mechanisms that take place in entry into JIA and that amplify uncertainty. The doubt is generated through mismatches between circle of acquaintance and physicians - especially General Practitioners (GPs) - and subjective experiences of the symptoms. It leads to less referral to the GP while inviting parents to engage themselves more intensively in the diagnosis and therapeutic process, resulting in the disease progressively taking a greater place in their life

According to our interpretation, the initial phase of floating in doubt sustains self-fuelling mechanisms that amplify the adverse impacts of the disease in everyday life. The doubt is generated through mismatches between circle of acquaintances and physicians and the subjective experiences of the symptoms. This doubt leads parents to engage in an active approach that will progressively anchor the place of the disease in everyday life. The initial period of uncertainty with a loss of confidence in healthcare together with the active involvement of parents tends to exclude the GPs from JIA monitoring (Fig. 2).

There are several levers of action to break these “mechanisms of uncertainty”: restore confidence and structure, re-engage GPs in the health monitoring framework, and offer guidance and support to parents actively approaching the disease. These self-energizing loops seem to be partially engaged once the pediatrician rheumatologist begins to take charge of managing the child’s disease.

Studies have already addressed the impact of disease on primary caregivers and family functioning in JIA and chronic diseases in general [13–16]. But the issue of the repercussions of these impacts has not been clearly addressed. Indeed, some papers highlight negative psychological effects, especially for the mothers [17–19], whereas others find no differences in quality of life and similar levels of anxiety compared to the general population [20–22]. For example, according to some controlled studies, families of children with JIA were found to show the same levels of adjustment as families with healthy children.

**Fig. 3:**
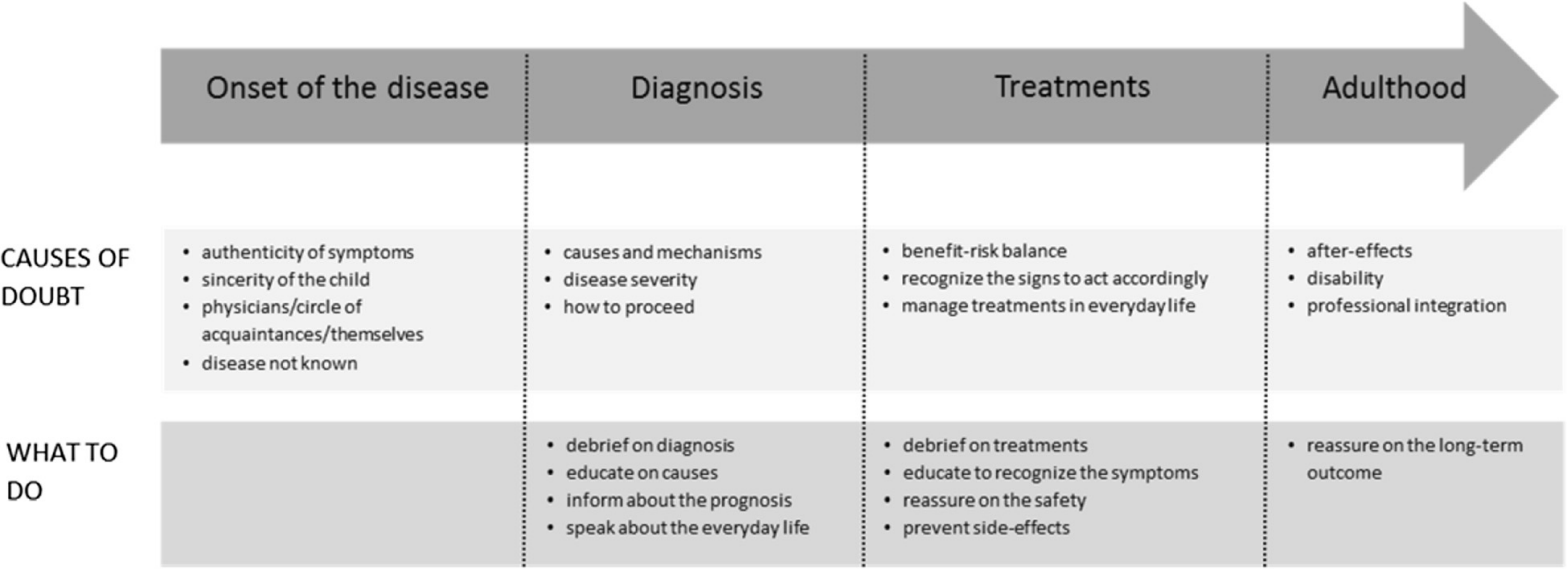
Parents’ doubts during the progress throughout JIA. Summary view of doubt in the different stages of progress throughout the disease, and keys on how to respond

Progress through this disease can be likened to a path for parents, which starts before diagnosis with different steps in the acceptance of the disease [23]. The consequences of these particular mechanisms that take root during the key phase of acceptance of the disease are little known in the short and long term. These mechanisms may sustain and amplify the complex emotional “rollercoaster ride” of ups and downs and highs and lows experienced by parents of a child with JIA [24]. They may influence parents’ experiences in every stage of progress throughout the disease, even if learning the diagnosis and more about the disease will bring some self-resolution of doubts (Fig. 3).

Full disclosure of all the information should be an answer to this doubt, but raw delivery is a double-edged sword [25]. Even if some parents want to know everything about JIA, the delicate balance between the benefits and negative consequences warrant tools to facilitate the communication process and answer the more or less medical questions that emerge outside the consultation visit window, reassuring them in the daily management of their child’s disease. This also enables the post-diagnosis consultation to be effectively split in two parts: first addressing the immediate questions about disease etiology and symptoms to ease parents’ anxiety, then later addressing questions about resources and support that inevitably arise. Even though these mechanisms are probably common to all chronic diseases, the subjective assessment of the child’s emotions and pain in a JIA setting makes this feeling of uncertainty more prominent [26]. JIA also suffers from its own specific issues: JIA is an under-recognized disease, determined only after progressively eliminating other possible diagnoses. From the early stages of the disease, the GP is prone to be excluded from medical monitoring of the children due to gaps in knowledge of the disease and the need for JIA-specific treatments. These features can explain why GPs are more at risk of being distrusted in JIA than in other diseases.

This study was designed as an overall assessment on how parents are engaged on the path to accepting a diagnosis of JIA for their child. As such, little can be concluded about special needs and specificities of each subtype of JIA. The study intended to recruit families with children affected by each form of JIA. However, in the qualitative part, only one child presented a systemic arthritis. In the systemic-onset subtype, the pre-diagnosis period is different from other subtypes, as it generally involves initial hospitalization and a shorter lag from onset to diagnosis. However, the main findings of our study (difficulty in understanding the cause of the disease, distrust of the GP, uncertainty over the future, self-delivery of medications) also apply to this subtype.

For this study, we chose a sequential design. Quantitative findings were used to complement the initial exploratory qualitative data. The methodological approach with verbal testimonies of lived experiences can lead to amplified and/or distorted recall of memories and make it difficult to recover a complete picture together with whole context in which experiences took place. We worked with a closed-ended questionnaire without neutral values. This could have forced parents to make a choice that reflects a possibly more settled opinion that in reality. Although the total response rate (75 %) may be considered acceptable for a self-administered questionnaire, the missing values rate (nearly 30 % in some cases; Table 3) may be considered a limitation.

The main objective of this study was not to be representative of the whole population, nor to try to quantify the frequency of the phenomena, but to identify benchmarks for parents in the path from experiencing the initial JIA symptoms in their child through to the pre- and immediate post-diagnosis period. The use of initial interviews to highlight the real-life experience of parents and the emotional impact and repercussions of the diagnosis allowed us to formulate more relevant questions to uncover details important to understanding these phenomena.

Taken together, our data support the implementation of a specific process dedicated to breaking the news of JIA to parents. The parents need an appropriate assistance with close monitoring from the outset, as well as more specific guidance, not only on the changes to everyday life but also the causes and mechanisms of disease. As such, the term “idiopathic” fuels the confusion. From this standpoint, as it is well established that all categories of JIA entail immunological pathology, a more precise name for the disease, such as “inflammatory arthritis” could be more descriptive than the current, more obscure “idiopathic” arthritis.

Given the lack of published references in this field, we propose an 8-point “memo” of key points to guide the delivery of diagnosis of JIA:

1. Explain the immunological background of the disease
2. Debrief of the medical trajectory
3. Develop communication facilities such as hotlines and information fliers.
4. Re-engage the local-area medical network (involve GPs providing information on JIA)
5. Take sibling(s) into account
6. Offer psychological support and counseling
7. Start education about the importance of compliance and adherence to treatment
8. Second consultation within 15 days

## Conclusion

Parents in the process of learning that their child has JIA are prone to 3 simultaneous phenomena:

1. they are incited to doubt everything
2. while the disease tends to take over their life,
3. but the medical support they need is hampered by JIA-specific factors (JIA is either unknown or unrecognized by the GPs).

The lack of parental benchmarks during the onset of JIA justifies a specific process for delivery of the diagnosis, with specific initial monitoring to enable both parents and children to appropriate the disease in a calmer and more composed way, to be ready to work with the doctor for the next stages of setting clinical path and to minimize the damages often caused in the lead-up phase of frequent initial misdiagnoses.

## Availability of data and materials

The authors do not wish to share the data because the verbatims of the interviews are in French, and have not been exhaustively translated. Moreover, the ethics committee gave it consent for the study but not for the divulgation of these verbatims. The authors agree to share their quantitative data, but are still detailed in the manuscript.

However if requested, data can be communicated in accordance with these specific limitations (In this case, please contact: achausset@chu-clermontferrand.fr).

## Abbreviations

GP: General Practitioner
JIA: Juvenile Idiopathic Arthritis
